# To Stimulate Appetite

**Published:** 1892-11

**Authors:** 


### To Stimulate Appetite.

In a lecture on "Concentrated Food in the Treatment of Pulmonary Consumption" (*Pitts. Med. Rev.*), Dr. Thomas J. Mays says that much can be done to stimulate the appetite. For this purpose he often gives the following:

R. Acid Phosporic Dil .............................. ½ ounce.

Acid Nitro-Muriatic Dil .......................... ½ ounce.

Acid Sulhpuric Aromatic ....................... ½ ounce.

Tinct. Ferri Chloridi .............................. ½ ounce.

M. Sig.: Thirty drops in a half glass of cold, sweetened water during meals.

### The Natural Result.

A great enthusiast on the subject of chest-protectors recommended them to people on every occasion. "A great thing," he would say. "They make people more healthy, increase their strength and lengthen their lives." "But what about our ancestors?" he was asked. "They did not have chest-protectors, did they?" "They did not," was the triumphant reply, "and they are all dead now—all dead."

